# Spirit of the British and American Periodicals, &c.

**Published:** 1843-01-01

**Authors:** 


### Spirit of the British and American Periodicals.

### Dr. Mutter on Cicatrices resulting from Burns.*

Few subjects in surgery have excited more interest than the peculiarities of cicatrices resulting from burns, and the plan of treatment by which the deformities they occasion may be either alleviated or entirely removed. In pursuing this investigation it is necessary to direct our attention to several points.

1*st. The Nature of the Tissue to be divided or removed.*—Although the "*tissue of the cicatrix*," as it is termed by Dupuytren, always presents certain peculiarities distinguishing it from any healthy or natural structure, it yet exhibits modifications induced either by the *cause* or the *tissue* involved. The cicatrix of a burn, for example, can always be distinguished from that caused by sharp instruments; and again, both these from those resulting from cancer, ulcers, syphilis, &c. The cicatrix of an ulcer in mucous membrane, differs, too, from one taking place in the skin.

Nearly all formations of this tissue, however, when dissected, present much the same structure. We have, in the first place, a *delicate cuticle*, which may be detached by vesication or maceration. Beneath this inorganic tissue is a dense stratum composed of strong fibres, which cross each other at different angles, and are firmly bonded together. This is the true "tissue of the cicatrix" of Dupuytren, and the "inodular tissue" of Delpech, beneath which and the cuticle there is no deposit, as a general rule, of rete mucosum; hence the whiteness of cicatrices in the African. It contains no hair bulbs, nor sebaceous follicles, at least when the lesion is profound, and although furnished with both nerves and blood-vessels, is usually less perfectly organised than the parts whose loss it supplies.

Lying under this tissue, we find a dense laminated structure composed of the original cellular substance, which binds down the cicatrix, and offers in many cases the chief obstacle to the success of our operations. This is especially the case in severe burns: and whenever such adhesions exist, we must anticipate and be prepared for, most extensive dissection, if an operation be attempted.

Another difficulty occasionally, but very rarely, presents itself in cases dependent upon burns—namely, the vascularity of the cicatrix. Whenever this tissue is red, sensitive, soft, and moveable, we may fear hæmorrhage; and this condition will, therefore, always render our prognosis, so far as loss of blood is concerned, more unfavourable than when the parts are pale, firm, inelastic, and adherent.

2. *The Thickness or Profundity of the Cicatrix.*—When the integument merely is involved, the cicatrix is for the most part elevated, thrown into bands, moveable and soft, the fascia beneath not being contracted. The motions of the subjacent parts are also normal, and hence, although the deformity may be considerable, the positive inconvenience is comparatively slight. In such a case the prognosis is favourable, and the operation required, much less severe than under other circumstances. When, on the other hand, not only the integument, but the superficial fascia, cellular tissue, and muscles are attached, the inodular tissue is irregular, dense, thrown into hard ridges, immovable or nearly so, and the parts which it unites are displaced, or, as in the case of openings and cavities,

* American Journal of Medical Sciences, July, 1842.

obliterated, the prognosis is very unfavourable, and the operation required extensive and severe. This condition must not be confounded with that contraction of the fascia superficialis sometimes accompanying cutaneous burns, but often the result of other causes, many of which are inappreciable.

3. *Location of Cicatrix.*—The location of the cicatrices will also modify the prognosis and treatment. When vital or highly organized regions are involved, great caution must be exercised before attempting an operation, and when such a procedure is deemed advisable, the patient should be warned of the probable risk. In deep cicatrices of such parts, there is less of danger of hæmorrhage than would be imagined, because the bloodvessels, especially the veins in the vicinity, are obliterated and converted into fibrous cords.

4. *Extent of Cicatrix.*—The wider and more extensive this is, the more difficult will it be to effect its removal. And we are hardly justified in the performance of an operation, unless we feel certain of obtaining a less deformed cicatrix than the one we wish to remove.

Dupuytren gives some very excellent advice relative to extensive operations on cicatrices: when, for instance, adhesions between the arms and thorax, or thigh and pelvis, are to be divided, he cautions us not to complete the operation at once, but to proceed by fractions, and let the wound of one operation heal, before we attempt another. In this way we avoid the dangerous consequences which would follow so large a wound as would be requisite to separate the parts at once.

Another good rule is, to be certain, before attempting any operation, that the limb retained in a faulty position is not incapable of being brought into a better one: if anchylosis, alterations of articular surfaces, or atrophy of the member is present, no operation should be attempted.

5. *Age of Cicatrix.*—Dupuytren advises "that no operation should be attempted until several months or even years have elapsed since the healing of the wound." He believes that we run great risk of exciting inflammation and ulceration in the part, and, moreover, that inasmuch as the *disposition of the cicatrix to contract* is not lost for a long period after its complete formation, we do no good by an operation which may indeed excite in this disposition a new energy. The older, then, the cicatrix, according to him, the better, so far as an operation is concerned. This advice is at variance with that of some other surgeons, but it is nevertheless as a general rule the safest to adopt. This is especially the case where the inodular tissue is superficial, and curable by simple incisions, followed by extension and pressure sufficient to keep the edges of the wound separate from each other. Of course, if the cicatrix is so situated as to interfere with the comfort and convenience of the patient, it may be proper to deviate from this rule, and operate as soon as possible.

6. *Peculiar Deformity of Cicatrix.*—The power with which these cicatrices contract is well known, but is sometimes overlooked in the desire for an operation. Mr. Earle has known it sufficient to bring the shoulders towards one another by a partial absorption of the clavicles. Cruveilhier mentions a case in which the carpus was luxated from the radius by a cicatrix on the back of the hand; and many other deformities might easily be cited. In these cases, of course, no ordinary operation would prove of any service.

When, therefore, the original shape and function of a part have been destroyed, we should never operate, unless there is a prospect at least of removing the deformity. There are cases in which we must be content with this, while the loss of the function is an evil for which there is no remedy.

Diversified as are the deformities from burns, Dupuytren is of opinion that they may all be referred to five classes :—

1. Those in which the cicatrix is too narrow.
2. Those in which it is too prominent.
3. Those in which it has formed extensive adhesions.
4. Those in which a cavity has been obliterated.
5. Those in which an organ or organs have been destroyed.

*Operations.*—It must be obvious that as the cicatrices present a great variety of shapes, occupy different positions, and penetrate to different depths, the operations for their removal must be modified to suit the case.

1. *Narrow Cicatrix—Incision.*—Suppose, for instance, the deformity to consist in the formation of a narrow band of inodular tissue, what operation is most likely to relieve it? Some surgeons recommend *incision of the band,* as performed by the ancients, whilst others tell us that it is almost if not altogether useless, and what is worse, that it even increases the difficulty, each incision in cicatrizing, shortening the band more and more. The latter view, though in the main correct, is not absolutely true, for entire relief has, in many cases, been obtained by means of incision and pressure. Much depends on the duration of the case, and the depth to which the cicatrix extends. If of long standing and sufficiently deep to involve the fascia superficialis, the probability is, that the operation will fail, owing to the contraction of the muscles which thus acquire a new sphere of action, and to the adhesions of the fascia. In recent and superficial cicatrices, however, the plan will answer, and in its execution, there are three indications to be observed.

1. The incisions are to be made at several points, and completely through the tissue; a scalpel or bistoury is the instrument to be employed.

2. The parts are then to be separated from each other, and placed at once, if supple and yielding, in their natural position; if rigid, a slow and gradual extension is to be kept up by splints and bandages until our end is accomplished.

3. Extension must be kept up for some time after the completion of the cicatrix, and if new fascia or bands form, they must be divided.

2. *Prominent Cicatrix—Excision.*—When the cicatrix is too prominent, there is rarely any unnatural contraction of the parts beneath, the elevation being almost entirely confined to the skin, and, consequently, all the operations in use are limited in their extent to this tissue. The one most to be relied on, is that proposed by Dupuytren, in which there are three things to be observed.

1. The projecting point is to be sliced off on a level with the skin.
2. The edges of the wound are to be kept apart by appropriate machinery.
3. The surface of the wound is to be frequently cauterized with argent. nit., so as to keep it rather below the level of the integuments.

3. *Extensive Adhesions.*—When the deformity consists in adhesions by which parts are approximated that should remain separated, or others separated that should remain in contact, numerous operations have been proposed.

Dupuytren's practice was as follows:—

After having divided the adhesions, he dissected them freely to beyond their origin. He then drew the parts asunder, and maintained methodical and constant pressure on the point whence the cicatrix must proceed, which is always at the angle of union of the parts.

This plan succeeds in some cases, but very often fails.

Another operation consists in cutting out the cicatrix, and bringing the edges of the wound together, so as to cover the raw surface from which the cicatrix was removed; and then extending the part by splints and bandages, and keeping them in this condition while cicatrization is going on, and for some weeks afterwards.

By this plan the contraction takes place in a lateral direction, and not in the long axis of the part operated on, and the cicatrix is soft, linear, moveable, and as extensible as natural integument. Whenever practicable, this operation is probably as good as any that can be devised, but in some cases it is impracticable.

Dr. Mütter has succeeded by slightly modifying this operation in curing a very extensive cicatrix, involving the arm and fore-arm, by which the whole member was rendered useless. After cutting out the cicatrix, he found it impossible to draw the edges of the wound over the raw surface; it then occurred to him that the only method by which success could be secured would be that which he had frequently resorted to in the operation for cleft palate, when there was difficulty in approximating the edges of the cleft, and which consisted in making *lateral incisions* at some distance from the edges of the tissue to be displaced. In doing this, and then drawing the edges of the original wound together, the raw surface could be completely covered; then dressing the two lateral wounds with warm-water dressing, made them unite by granulation. The operation succeeded perfectly, and may be had recourse to in many similar cases.

Another plan, the principle of which was clearly recognised by Celsus, which Dr. Mütter has tried in extensive cicatrices about the neck, without, however, deriving much benefit from its employment, consists in making an incision through the integuments at some distance from the origin of the cicatrix, in other words, in perfectly sound skin; in dissecting up the skin and cicatrix as far as possible, without making any new incisions in the skin itself; and then separating the divided parts, so that the cicatrix slides from its original position, leaving a raw surface to heal on granulation.

The operation is severe, and though sometimes useful, is not much to be relied on in cases of extensive contraction.

The operation which, of all others, Dr. Mütter considers as most entitled to confidence, especially in cases of cicatrices of the neck, cheek, eyelids, nose, and lip, is that in which "autoplasty" is brought into service. In all such operations we are governed by the same principles, and pretty much the same mechanical details.

They consist in, dividing the cicatrix, so as to produce a raw surface in some part of its extent, or else cutting it out entirely; in applying to this raw surface a piece of healthy skin taken from the neighbouring parts; in attaching this skin by suture to the margins of the wound in which it is inserted; in approximating the edges of the wound from which the skin has been removed; in separating, by appropriate agents, the parts too closely approximated, and keeping them in this condition, some time after the flap has united: and in applying oleaginous frictions and motion to the new made parts to give them flexibility and softness.

Many shocking deformities from burns have been relieved by the performance of operations conducted on this principle; for example, the eyelids, the cheek, the nose, and the lip, have all been restored; but Dr. Mütter claims the merit of having first performed an operation of this kind for the relief of extensive cicatrices of the throat.

In very extensive cicatrices of the neck, it may be well to modify the operation so as to take a flap from each side, by which means the risk of a very large single flap will be avoided.

4. *Cicatrices complicated with Obliteration of Cavities.*—Where the cicatrix produces partial or complete obliteration of a natural opening, as the mouth,

&c. incision of the angles, and the introduction of tents larger than the natural opening, will occasionally do good, but for the most part all such attempts fail, and it becomes necessary to perform the operation recommended by Dieffenbach.

5. *Cicatrices complicated with Loss of Organs.*—Where organs are entirely destroyed, nothing short of a "plastic operation," the aim of which will be the construction of an organ as much as possible like the original, offers the slightest prospect of benefit to the patient.

---

### Observations on Pericarditis and Endocarditis, and on the best Mode of Treatment in these Diseases. By E. O. Hocken, M.D. &c.

The main features, of this Paper may be condensed into the following propositions:—

1. That the heart is always exempt in gonorrhœal rheumatism. 2. That, in the atmospheric form, it suffers only in the articular variety, but that no stage of the rheumatism, especially where the synovial membranes are unaffected, should be considered too early for minute and frequent investigation of the cardiac region by the stethoscope, as heart-disease sometimes precedes the more obvious symptoms, nor should any period short of complete recovery be thought too late to search for, rather than be surprised with their outbreak. 3. That as the heart suffers in the proportion of one-half or one-third of the cases of acute articular rheumatism, and as these diseases almost invariably lay the foundation of future disease and future death, although rarely proving immediately fatal, that it is the bounden duty of the practitioner constantly and carefully to watch for and search after the signs which lead us to diagnose their very commencement; since all our experience tends to prove that these diseases are never *cured* unless stopped *at once*. 4. That there are auscultatory signs which precede the formation of a bruit, and the to and fro sound of friction, as trustworthy and as diagnostic, when occurring de novo, as could be a loud bellow's murmur, or a bruit de scie. 5. That these stethoscopic signs stand in the following order:—1st. An increased and abrupt impulse of the heart. 2nd. An increased frequency in the actions of the heart. 3rd. A lengthening and roughening of the sounds of the heart, chiefly or entirely marked in the first sound. 4th. A diminution of the diastolic period, chiefly marked in the period of repose. 5th. The commencement of a distinct bruit. 6th. A roughening of the to and fro motions of the heart, without a distinct bruit de frottement. 7th. That the stethoscopic signs are combined with a thrill or vibratory impulse in the pulse. 8th. That more general symptoms are most deceptive guides, at the same time that one symptom, when present, is very characteristic and one of the earliest indications of any implication of the heart, viz. the peculiar expression of anxiety which the countenance so generally betrays, and which is foreign to the mere course of the rheumatism. 9th. That the inflammation seems to commence in, and to be principally confined to, the true fibrous tissues, secondarily involving the serous membranes in contact with them. 10th. That when a bruit de frottement is fully formed it never terminates but in complete adhesion of the roughened surfaces, or in the death of the patient—probably depending upon the quantity of solid lymph thrown out in these cases. 11th. That when diagnosed and treated even at the commencement of a bruit and friction sounds, the future prognosis is very unfavourable. Watson, Latham, &c. all concur that, under these circumstances, a large proportion will seem to recover, but that the recovery is so far unreal that it involves the germs of future destruction. 12th.

That every case of acute articular rheumatism, especially when the synovial membranes are free from implication, and the disease is erratic, threatens imminent peril to the well-being of the heart, and on this account should always be treated in expectancy of such an occurrence. 13th. That this treatment should consist of moderate venesection, avoiding all risk of shocking the system —calomel, in doses of ten or twenty grains, mainly with the object of its peculiar action as a purge, every twelve hours, to the fourth or fifth dose, with half a grain of opium, followed, if necessary, by some castor oil—subsequently calomel in smaller doses, keeping the system so far under its influence, that we can rapidly produce soreness of the gums when we will it by modifying the dose, and yet so far from it, that all the symptoms of salivation are absent, only the constitution is on the point of their production,—one or two grains of opium twice or thrice a day. 14th. That on the occurrence of the first stethoscopic signs of heart-disease, blood should be drawn to the amount each case may suggest, avoiding syncope, and the calomel increased, so as to produce moderate salivation, *as soon as possible*, viz. one-grain doses with one-eighth of opium every hour.—*Lond. and Edin. Monthly Journ. of Med. Science.*—Sept. 1842.

---

### Dr. Mettauer's Case of Artificial Penis.*

Mr. ———, æt. about 19 years, of good general health, though much depressed in mind by the mortifying deformity under which he laboured, came under the Dr's. care in the Autumn of 1841. His penis was greatly elongated, measuring eight inches from the scrotum to the extremity of the glans in the non-erected state. The anterior three-fifths, much dilated laterally, with great expansion of the corresponding portion of the urethra, was perfectly flaccid and non-erectile. The urethra in this part was capable of containing two ounces of fluid, and the cavity which it formed was bounded anteriorly by the concave glans; posteriorly by the rough granular surface of the pubic two-fifths, or stump of the organ; and laterally by walls formed of the urethra greatly dilated, the elastic ligament, and the integuments. The pubic portion, or stump, constituted two-fifths of the penis, was well-formed, capable of erection, and terminated abruptly, so as to form the pubic extremity of the cavity already described, with the orifice of the urethra projecting from its surface so as to form a kind of os tincæ looking into that cavity. Low down in the perineum, about ten lines anterior to the verge of the anus, was a small orifice by which the greater part of the urine escaped. The urethra about eight lines in extent anterior to this orifice was nearly impervious, and transmitted only a few drops of urine during micturition. On the central part of the expanded, pouch-like portion of the urethra, there existed at this time a fistulous opening, which had been formed artificially, and through which a few drops of urine would occasionally appear. From the meatus, a muco-purulent discharge frequently escaped, especially when the urine was forced to take that direction.

Notwithstanding this deformity, strong sexual desires existed. The testes were perfectly formed, and of large size. Dr. Mettauer being of opinion that it was practicable to correct the deformity, so far, at least, as to place the organ in a condition favourable for sexual intercourse, the following plan was adopted, and, as the sequel will show, it occasioned the perfect correction of the malformation.

The first aim was to transplant the glans upon the erectile stump. To accomplish this, the pouch was laid open in its whole extent by an incision along the rapheal line. A belt was then removed from the interior of the cavity seven lines

---

* American Journal of Medical Sciences. July, 1842.

in width entirely round the base of the glans, and quite to that organ, so as to leave that part of the wall of the pouch to consist only of integument and cellular membrane. A like belt was removed from the inferior portion of the pouch, quite down to the circumference of the face of the erectile stump, which was then carefully denuded in every part of it. After the blood had been carefully sponged away, the glans was placed with great care upon the face of the stump, taking care that the denuded margin at its base should exactly correspond with the circumference of the opposed surface of the stump. A short bougie was then passed into the meatus, and carried along the urethra of the stump, nearly to the contracted portion of it, in order more easily to retain the glans in its proper position. Thus arranged, the glans was firmly connected to the erectile stump by eight points of the glover's suture, taking care that ample space between them was allowed for the free passage of blood to the glans. When the sutures were tightened, they fixed the glans most perfectly and securely on the erectile stump, and imparted to the organ, thus modified, an improved condition highly gratifying!

This procedure necessarily shortened the penis, and required that the tegumentary intermedium which had been left, should be inflected upon itself, so as to form a loop-like body on the dorsal and lateral portions of the organ immediately behind the glans. This was unavoidable in order to supply the glans with a sufficiency of blood for its nutriment, but to remedy as much as possible the inconveniences which it occasioned, about one half of the band which it formed, was removed by the scissors, taking care not to disturb the sutures.

Little inflammation followed the operation, and by the third day free suppuration was established throughout all of the ununited cut and denuded parts. At the end of the twelfth day the glans was found to have united firmly to the stump, the margins of the intermedium, however, did not heal over until the twentieth day, and fully three months elapsed from the date of this operation before all inflammatory tenderness of the parts involved had subsided.

It was then resolved to remove the unsightly fold of integument left for the support of the gland, which was accomplished by first rapidly excising the parts with a strong pair of scissors down to the depth of their union, and then cautiously dissecting away the remaining portion, taking care to leave no more integument than would suffice to render the organ comely. As soon as the superfluous textures were dissected away, the margins of the skin were carefully approximated, and retained by sutures. At the end of a fortnight, the sutures were cut away, and the part found to be firmly united and well.

The penis now presented a very natural appearance, and was fully two inches in length during the non-erected state, measuring from the scrotum. The glans, too, which had lost its sensibility from the moment the intermediate skin between it and the stump was divided, had in some slight degree at this period recovered its feeling, and manifested a decided erectile blush, and some expansion from friction. In the erected state, the penis measured nearly four inches and a half in length, and presented in all respects a most natural appearance.

Several months elapsed before it was deemed prudent to attempt any operation for restoring the urethra to its pervious and proper condition, as well as for closing the opening in the perinæum. At the end of this time, all things being favourable, the operation was performed in the following manner.

A curved probe was passed into the perineal opening, with the point firmly pressed in the direction of the glans into the cul-de-sac at the termination of the inferior urethra, which was entrusted to an assistant to be held steady. A long trochar was then introduced into the meatus, and carried quite down through the anterior or superior urethra to its cul-de-sac, with the concavity to the symphysis, and the lancet retracted within the canula, held and directed with the right hand. The blunt extremity of the canula was now pressed firmly into the bottom of the cul-de-sac, and after giving it the proper direction, the lancet was projected from its concealment about five lines, and immediately retracted within the canula. The canula was now pressed onwards so as to fill the part incised quite to the bottom, and the lancet again projected, and in this manner the operation was repeated, until the extremity of the canula entered the inferior portion of the urethra. The trochar was then withdrawn, and a gum catheter of proper size introduced into the bladder.

The opening in the perineum, from its external margin down to the tube, was then touched with lunar caustic, and the eschars carefully scraped off with a small, delicate scalpel, so as to expose a new denuded surface; the opening was then closed with two points of the interrupted suture, inserted from within outwardly down to the tube, and fully eight lines from the margins. Directions were given that the bladder should be evacuated every second or third hour, to guard as far as possible against the passage of urine around the tube: an accident which might occur in the event of a large accumulation of water in the bladder taking place.

In this situation the case was suffered to remain for five days, at the end of which period the tube was carefully removed, and the parts found to be in a most favourable condition for a speedy and perfect cure. The urethra suppurated freely, but not too much so, and the margins of the perineal opening seemed firmly united. A fresh tube was introduced and kept in the passage for three days more; after this it was only introduced during urination, to prevent, if possible, any stress from the flow of water along the urethra upon the newly-closed perineal opening. On the twelfth day the sutures were cut away and the margins of the opening found to be firmly and perfectly united.

The patient was advised to introduce a bougie along the newly-formed passage, at least once a day for a year, and after that period to employ it occasionally to prevent the contraction.

The urethra was now free from all tenderness, and transmitted the urine from the bladder in a bold and full stream.

Thus modified, there is little doubt but that the penis will prove useful for all purposes, and compensate the patient for the pain and suffering he endured from the different operations performed for his relief.

